# The development of a nursing subset of patient problems to support interoperability

**DOI:** 10.1186/s12911-017-0567-5

**Published:** 2017-12-04

**Authors:** R. A. M. M. Kieft, E. M. Vreeke, E. M. de Groot, P. A. Volkert, A. L. Francke, D. M. J. Delnoij

**Affiliations:** 1 Dutch Nurses’ Association, PO Box 8212, 3503 Utrecht, RE Netherlands; 2 Nursing Healthcare Innovation Technology Platform of the Dutch Nurses’ Association, PO, Box 8212, 3503 Utrecht, RE Netherlands; 3 Nictiz, Dutch National release center of SNOMED CT, PO Box 19121, 2500 The Hague, CC Netherlands; 4 Nictiz - Netherlands Institute for Health IT, PO Box 19121, 2500 The Hague, CC Netherlands; 5 Netherlands Institute for Health Services Research (NIVEL), PO Box 1568, 3500 Utrecht, BN Netherlands; 6 Amsterdam Public Health Institute/VU Medical Center, Department of Public and Occupational Health, Van der Boechorststraat 7, 1081 Amsterdam, BT Netherlands; 7 National Health Care Institute, PO Box 320, 1110 Diemen, AH Netherlands; 8 Professor of Transparency in Healthcare from the Patient’s Perspective, Tranzo, Tilburg University, PO Box 90153, 5000 Tilburg, LE Netherlands

**Keywords:** Interoperability, Nursing, Problem list, SNOMED ct, Standardisation

## Abstract

**Background:**

Since the emergence of electronic health records, nursing information is increasingly being recorded and stored digitally. Several studies have shown that a wide range of nursing information is not interoperable and cannot be re-used in different health contexts. Difficulties arise when nurses share information with others involved in the delivery of nursing care. The aim of this study is to develop a nursing subset of patient problems that are prevalent in nursing practice, based on the SNOMED CT terminology to assist in the exchange and comparability of nursing information.

**Methods:**

Explorative qualitative focus groups were used to collect data. Mixed focus groups were defined. Additionally, a nursing researcher and a nursing expert with knowledge of terminologies and a terminologist participated in each focus group. The participants, who work in a range of practical contexts, discussed and reviewed patient problems from various perspectives.

**Results:**

Sixty-seven participants divided over seven focus groups selected and defined 119 patient problems. Each patient problem could be documented and coded with a current status or an at-risk status. Sixty-six percent of the patient problems included are covered by the definitions established by the International Classification of Nursing Practice, the reference terminology for nursing practice. For the remainder, definitions from either an official national guideline or a classification were used. Each of the 119 patient problems has a unique SNOMED CT identifier.

**Conclusions:**

To support the interoperability of nursing information, a national nursing subset of patient problems based on a terminology (SNOMED CT) has been developed. Using unambiguously defined patient problems is beneficial for clinical nursing practice, because nurses can then compare and exchange information from different settings. A key strength of this study is that nurses were extensively involved in the development process. Further research is required to link or associate nursing patient problems to concepts from a nursing classification with the same meaning.

**Electronic supplementary material:**

The online version of this article (doi:10.1186/s12911-017-0567-5) contains supplementary material, which is available to authorized users.

## Background

Since the emergence of the electronic health record, nursing data is being recorded and stored digitally. Nurses constantly collect and analyse data from all contacts with the patient, whatever the setting. Data such as a weight or blood pressure is objective and does not include further (clinical) interpretation on its own [1]. Data only becomes meaningful if it can be interpreted within a certain context. For instance, weight becomes relevant when a patient has lost a considerable amount of weight in a short time or when a patient is suspected to have anorexia. When data is placed within a context, it is defined as information [1].

Record-keeping is important, because this information is the basis of communication between nurses and patients and other professionals. It is also the basis for planning care, making decisions about interventions and evaluating the results [2]. In addition, the need to exchange or reuse information within and across different healthcare settings has been increasing over recent years. Patients are hospitalised for shorter durations; their recovery is shifting from hospital to an ambulatory care setting, primary care or home care.

In order to share and exchange information without risk of misinterpretation, nursing data needs to be unambiguous. There is a growing body of literature that recognises the importance of this issue. The studies by Park and Cho [3], Westra et al., [4], and Randorff Højen and Rosenbeck Gøeg [5] emphasise that the words i.e. terms and meaning that nurses need for record-keeping should be defined consistently using terminology that facilitates reuse. However, several studies looking at nursing documentation have shown that a great variety of terms are used across and within different healthcare settings: locally preferred terms [3] as well as multiple terminologies or classifications (such as the international classification of functioning and disabilities (ICF) [6], Omaha System [7] or the classification for nursing diagnosis (from NANDA International; NANDA-I); interventions (the Nursing Intervention Classification; NIC) and nursing outcome (Nursing Outcome Classification; NOC) (NNN) [8]).

This also applies to the Netherlands, where healthcare organisations are currently shifting from a paper-based to an electronic health record system. According to the national eHealth monitor among nurses, 84% of hospital-based nurses record their nursing data digitally, in contrast to only 40% of nurses working in home and primary healthcare organisations, who in some cases record data both digitally and on paper [9]. Both locally preferred terms and different classifications (e.g. the Omaha System, Nanda-I diagnosis or ICF) are integrated in the electronic health record but without consistency across different nursing settings. Difficulties arise when nurses share and exchange information with others involved in the delivery and continuity of nursing care; this is also known as the interoperability issue [10–12].

In the last decades of the twentieth century, the diversity in nursing information was already discussed by Dutch researchers and work has been undertaken to investigate the need for one standardised nursing language [13–15] or to collect standardised nursing data to analyse and compare nursing data across populations, settings, geographical areas and time [16]. Despite these efforts, there is still a diversity of nursing information, impeding the exchange and reuse of nursing information within and between healthcare sectors [17]. Currently, the Ministry of Health, Welfare and Sport, stakeholders (e.g. the national competence centre for standardisation and eHealth (Nictiz), the Netherlands Federation of University Medical Centres and the Dutch Hospital Association) and professional organisations (e.g. the Dutch Nurses’ Association, hereinafter referred to as the collaborating parties), collaborate to develop, construct and maintain unambiguous data for professionals involved in patient care, including nurses [18–21]. This means that nursing and other professionals should transform various (nursing) data using different coding systems into a single common format to allow comparison and exchange of data. One key aspect in the development is one standardised language for all professionals. The preferred terminology for professionals in the Netherlands and many other countries (e.g. United States, United Kingdom, etc.) involved in patient care is SNOMED CT. This terminology contains more than 300,000 concepts. Each concept encapsulates a clinical thought or idea, for instance a patient problem [22]. A concept has one or more terms that must unambiguously represent the meaning of the concept, such as ‘pressure ulcer’. A concept can have corresponding synonyms allowing local preferences or dialects (e.g. pressure sore or contact ulcer) or to express terms in different languages (e.g. the Dutch term *decubitus* or the Spanish term *úlcera por decúbito*). The concept has the same covering details consisting of a single unique code or identifier 399,912,005, allowing professionals to exchange and reuse information [23] (http://browser.ihtsdotools.org).

Because a terminology can contain an enormous number of concepts, subsets are developed to ensure appropriate use in daily practice. Subsets consist of specific concepts selected from the core set representing a particular context, for instance patient problems in nursing practice [24].

Subsets focused on patient problems, also called subsets of patient problems or catalogues, have been discussed in several studies [25–27]. These studies pointed out the importance of the clinical domain being covered. Using a pre-existing national survey panel (Nursing Staff Panel; see http://www.nivel.nl/panelvenv), Dutch clinical nurses were asked to indicate which patient problems they most frequently encountered in daily practice, as well as the influence nurses said they had on these problems [28]. This resulted in an overview of patient problems reflecting Dutch clinical nursing practice domain across healthcare settings (version 0.1) as shown in Additional file 1: Overview of patient problems (level of occurrence compared to level of reported influence). Nevertheless, these patient problems and their meanings need to be specified in detail to enable consistent and accurate use by nurses in clinical practice.

The aim of this study is to develop a national nursing subset of patient problems based on the SNOMED CT terminology to assist interoperability, using the overview of patient problems mentioned earlier as a framework.

This developed subset of patient problems will benefit standardisation and consistent use of nursing information in electronic health records, and improve communication between nurses and other professionals within and across different healthcare settings.

### Research question

Which SNOMED CT concepts cover patient problems frequently encountered in Dutch nursing practice?

### Conceptual framework

**Fig. 1:**
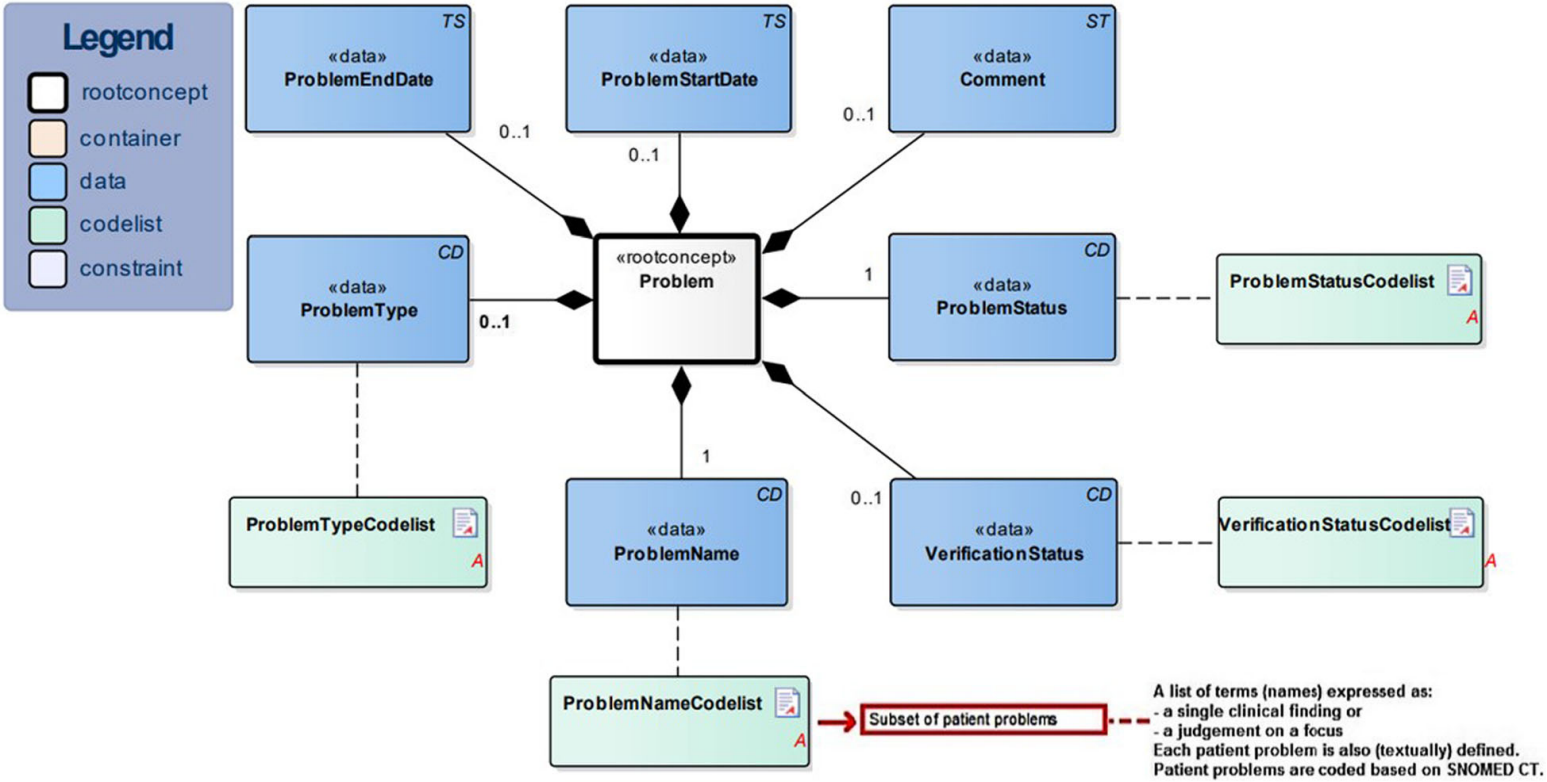
Clinical building block for patient problems (diagnosis) version 3.0 (01–05-2016)

The construction of unambiguous (nursing) data has been based on an information model also known as a detailed clinical model or a clinical building block [29] and is established by the collaborating parties [30]. A single clinical building block describes a certain clinical concept and the characteristics thereof that should (required data items) or could (optional data items) be recorded and in what way (e.g. physical quantities or predetermined coded values). One such clinical building block describes patient problems (i.e. diagnosis, Fig. 1) including the required data item “problem name”. This data item defines the problem based on a predetermined code list: in the context of nursing practice, it can refer to the nursing subset of patient problems developed in this study. More detailed information about clinical building blocks can be found on: https://zibs.nl/wiki/HCIM_Mainpage.

The nursing patient problems are constructed in line with the ISO 18104 standard. This standard is established by the International Organisation for Standardisation Technical Committee (ISO/TC) and describes a set of shared characteristics for constructing nursing diagnoses (e.g. clinical finding, focus, judgement) [31]. It means that each nursing patient problem can be expressed as a single clinical finding (e.g. anxiety, pain) or as a judgement on a particular focus (e.g. walking disability). A judgement is an opinion or finding related to a focus (e.g. disability, ineffective). A focus is an area of attention (e.g. walking). Patient problems are coded based on SNOMED CT.

Clinical building blocks for patient problems let healthcare professionals, including nursing professionals, describe and report their practice in a consistent manner and develop a single unified language [29]. For instance, medical specialists have developed a diagnosis thesaurus, a list of medical diagnoses based on the structure of the clinical building block and SNOMED CT [32]. For this study, a list of patient problems for nursing practice was developed based on the same SNOMED CT principles.

## Methods

**Fig. 2:**
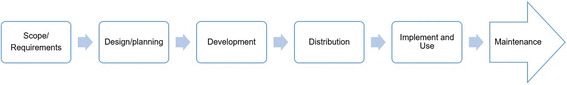
The process model from creation through to maintenance for SNOMED CT subsets (source IHTSDO [37])

Various studies have described methods for the development of terminological subsets [25, 27, 33–35]. Most of the studies referred to and used a process model (or aspects of one), meaning that a process from creation through to maintenance was described. The conclusion can be drawn that a process model seems to be promising as a method for developing a nursing subset. However, there also seems to be a lack of uniformity in the stages, approaches and techniques, so the process models have not yet been fully explored and are still evolving [36]. In this study we used the process model for the development and maintenance of subsets as part of the International Release of SNOMED CT as described by the IHTSDO [37] and the Dutch instruction ‘making a SNOMED CT subset’ derived from it and set up by Nictiz [38], which involved the following six stages: 1) Scope/Requirements; 2) Design/Planning; 3) Development; 4) Distribution 5) Implement and Use; 6) Maintenance. Figure 2 gives an overview of the stages. Each stage will be explained in the next paragraphs.

### Stage 1: Scope/requirements

In this stage, we defined the purpose of the subset and relevant requirements, such as the scope of content and the users. First an expert team was set up, consisting of a researcher (RK) and a nursing expert (EV), both with extensive knowledge of the structure and content of SNOMED CT, and two representatives of Nictiz (acting as the Dutch SNOMED CT Release Centre): the Terminologies Coordinator (PV) and a terminologist (EG).

The expert team identified the scope, which was to develop a national nursing subset of patient problems based on the SNOMED CT terminology to assist interoperability. The users of the subset were defined as clinical nurses working in various healthcare settings. Other existing subsets were then explored to evaluate whether they met the requirements. To our knowledge, two national SNOMED CT nursing subsets of patient problems have been developed, namely a United States (US) [25] and a Danish [26] nursing subset. Denmark developed a national homecare nursing subset of 80 concepts (not available online yet) building upon the US nursing subset [26]. Patient problems from the US nursing subset were retrieved from the ‘Unified Medical Language System’ (UMLS) Metathesaurus database, developed by the National Library of Medicine (NLM) [25]. The database contains concepts from various different classifications and terminologies. Queries were performed by the UMLS to collect patient problems from SNOMED CT and four nursing classification systems (the Omaha System, NANDA International, the Home Healthcare Classification (HHC) and the ICNP). The patient problems included were reviewed manually and discussed, resulting in 369 nursing problem concepts (https://www.nlm.nih.gov/research/umls/Snomed/nursing_problemlist_subset.html). Although this subset could be useful for building on, we decided to develop a new subset. The main reason for this decision was the findings of a previous study, ‘A nationwide survey of patient problem occurrence across different nursing healthcare sectors’, in which Dutch clinical nurses were asked to indicate which patient problems they encountered most frequently in daily practice, as well as the influence nurses said they had on these problems [28]. This resulted in an overview of patient problems (version 0.1) reflecting the Dutch clinical nursing practice across healthcare settings. Using this overview as a framework we could specify the patient problems as identified by nurses themselves. This approach differs from the US subset, which contains concepts from various different classifications and terminologies (regardless of their occurrence or the perceived level of influence).

### Stage 2: Design/planning

In this stage, we defined the composition, the involvement of participants, sampling and recruitment process. Focus groups with nursing professionals were held to determine which SNOMED CT concepts cover the patient problems (version 0.1). In order to recruit participants, an invitation to participate in the focus groups was sent in a digital newsletter from the Dutch Nurses’ Association (V&VN). This monthly newsletter was mailed to 70,000 members of the Dutch Nurses Association, giving information about the study as well as the registration process.

Sixty-seven nurses replied to the recruitment message in the newsletter and agreed to participate. We organised seven focus groups, using the following inclusion criteria:

□ Employed as nursing professional.

□ At least 2 years’ experience as a nursing professional.

□ Working in hospital care, residential care, psychiatric care, primary care or care for the mentally disabled.

Because the nurses were active in a variety of nursing practice contexts, patient problems could be discussed from different perspectives, which was necessary to determine whether the patient problems were comprehensive, unambiguous and acceptable in a broad nursing context. The expert team also took part in each focus group.

The focus group meetings lasted two and a half hours. The nursing expert (EV) led the meeting, explained the procedures, and introduced the method and the patient problems to be discussed. The terminologist (EG) identified and selected corresponding SNOMED CT concepts and ensured that the concepts were consistently and accurately applied in line with the SNOMED CT guidelines. The nursing researcher (RK) observed and monitored the process.

### Stage 3: Development

There is a variety of approaches for developing subsets, such as developing a new reference set or adopting, copying and adapting an existing reference set [37, 38]. In this study, developing a new subset was deemed appropriate, firstly because the development could build upon the existing overview of the study mentioned earlier in which 440 Dutch nurses had already participated [28] and secondly because the involvement of nurses could be maintained in order to improve backing and approval of the final subset.

The development process was set up in four phases [38]: 1) the selection of SNOMED CT concepts; 2) review and translation process with focus groups; 3) defining and modelling; 4) validation of the subset. This setup was based on the Dutch Nictiz instruction ‘Making a subset’ [38].

#### a) Selection of SNOMED CT concepts

The first phase comprised selection of SNOMED CT concepts by the expert team. The overview of patient problems (version 0.1) acted as a framework (Additional file 1). The patient problems (version 0.1) contained both Dutch and English terms. The expert team then selected and identified a matching SNOMED CT concept (or the nearest match) for each patient problem based on the term and definition in version 0.1. The concepts were selected from the core distribution of the International SNOMED CT Edition (January 2016 release) managed by SNOMED International and available online at http://browser.ihtsdotools.org/.

**Fig. 3:**
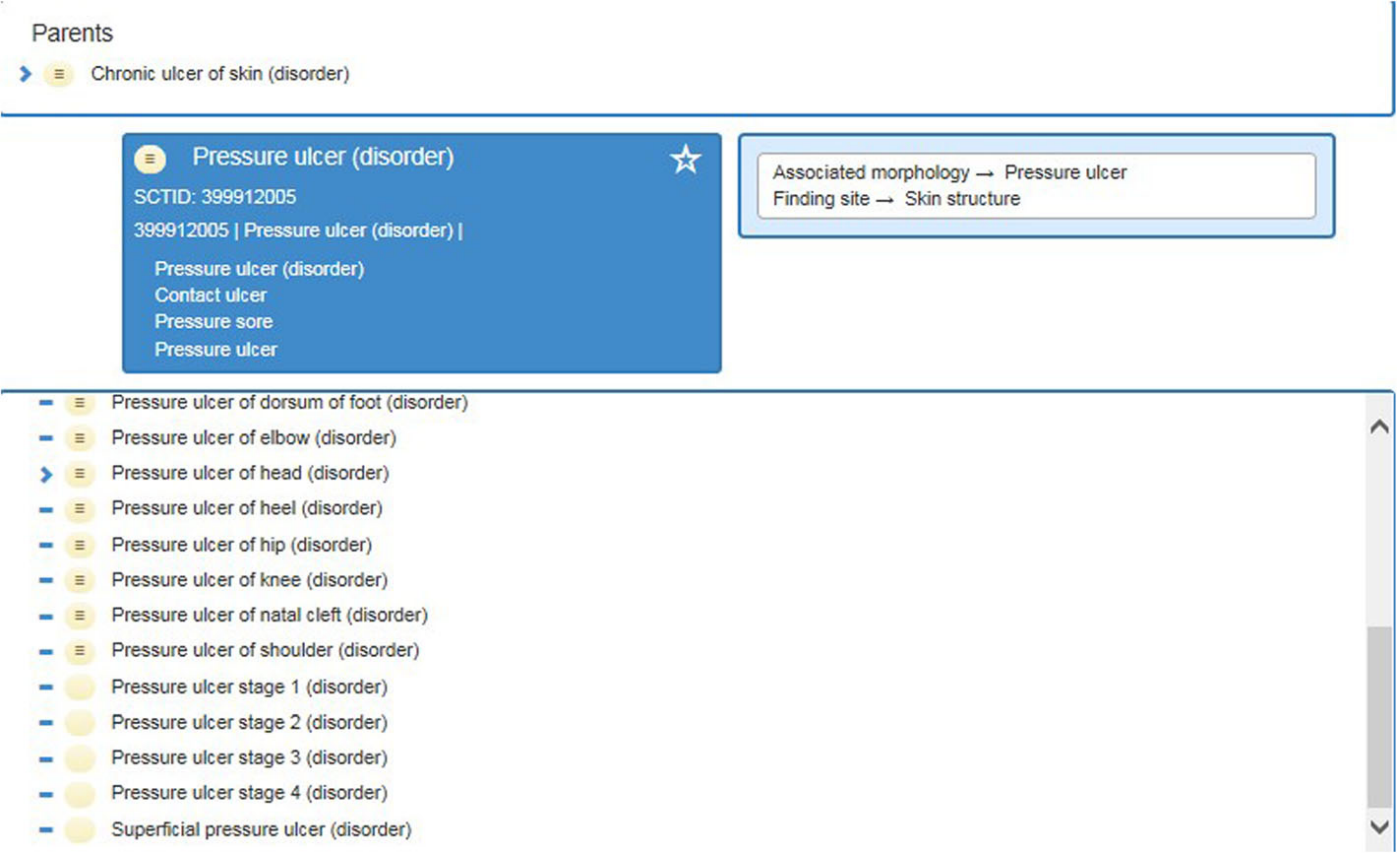
Example of the concept ‘Pressure ulcer’ in the SNOMED CT hierarchy (English edition, release version 20,160,131)

An example of the concept ‘Pressure ulcer’ from the core distribution of SNOMED CT is shown in Fig. 3. The concept has a unique numeric identifier (399912005) and equivalent synonyms (Contact ulcer, Pressure sore). Each concept is linked to a more general concept in the hierarchical structure, the so-called ‘parent’. In the example of a ‘Pressure ulcer’, the parent is ‘Chronic ulcer of the skin’. It is also possible to specify ‘Pressure ulcer’ in increasing detail. The specifications are referred to in the underlying hierarchy as ‘children’, for example ‘Pressure ulcer stage 1 and stage 2’ and so forth.

SNOMED CT concepts that were equivalent to concepts from the International Classification of Nursing Practice (ICNP) were preferred in order to ensure that the terms accurately represented the nursing domain. The ICNP is a formal terminology for nursing practice developed by the International Council of Nurses (ICN) [39]. SNOMED International and the ICN collaborated in order to harmonise both terminologies to increase interoperability and to encourage the use of terms as established by the ICNP [40]. SNOMED International and ICN developed an ICNP-to-SNOMED CT Equivalency Table for Diagnosis and Outcome Statements [41], meaning that each ICNP diagnosis included in the equivalency table has the same meaning as the SNOMED CT patient problems included (English edition, release version 20,160,131). The equivalency table was used to ensure that the SNOMED CT concepts matched consistently.

#### b) Review and translation process (with focus groups)

In the second phase, the patient problems plus matching pre-selected SNOMED CT concepts were reviewed and discussed. Each focus group discussed and reviewed an average of 12 patient problems. Both the patient problem from version 0.1 and the matching SNOMED CT concept were presented to the participants of each focus group. The SNOMED CT concepts were presented directly from the browser (see the example in Fig. 2) so that the hierarchy could be clarified by switching between different concepts and their parents or children if necessary. The participants discussed the preselected concepts using the following predefined questions:

- Is the term sufficiently comprehensive for electronic recording?
- Is the term unambiguous and understandable?
- Is the term professionally acceptable for nursing practice?

These questions were derived from the viewpoint of the Nursing Special Interest Group on the nursing contribution to quality assurance of SNOMED CT [42]. Nursing professionals participate in the Nursing Special Interest Group to advise IHTSDO on ‘the development, validation, uptake and implementation of SNOMED CT and related products’ [43] (p. 4).

Each concept had a SNOMED CT term derived from the English edition (release version 20,160,131). The terms were translated to Dutch following the SNOMED CT guidelines for translation [44]. The nursing professionals from the focus groups and the expert team were involved in the translation process. Nursing professionals confirmed that the preferred Dutch terms corresponded to the terms used in their daily activities and were clinically acceptable.

The SNOMED CT patient problems included in the equivalency table have the same meaning as the ICNP diagnosis. We were therefore able to validate the translation process by using the Dutch catalogue from the International Classification of Nursing Practice (ICNP) [45]. The ICNP beta version, including terms and definitions, was translated (working in both directions) into Dutch by the Dutch Nursing Union (Nu’91) in cooperation with the ICN.

Once a focus group reached a consensus about a concept, the terminologist coded the selected concept. If a focus group did not reach a consensus about a concept, it was debated in another focus group until a consensus was obtained. If the groups found a concept to be either inconsistent or incomplete or if there were no appropriate concepts, requests for additions or changes to SNOMED CT or new concepts for it were submitted to the Dutch National Release Centre (Nictiz).

#### c) Defining and modelling

In the third phase, the expert team defined each SNOMED CT concept in Dutch (in SNOMED CT terms: ‘textually defining’). The (textual) definitions provide additional information about the intended meaning or usage of each concept. To ensure that the meanings of nursing concepts were reflected accurately, national Dutch guidelines were examined and the definitions available in them were used where possible. If no definition was available, the definitions of nursing diagnosis as established by the International Classification of Nursing Practice (ICNP) were used; these were also described in the Dutch ICNP catalogue [45]. If no definition was available in the ICNP catalogue, the definition from another classification was used (for instance the International Classification of Functioning and Disability).

After each focus group, the expert team broke the selected SNOMED CT concepts down into two items, a name and a textual definition. A SNOMED CT concept could be expressed as a single clinical finding or as a judgement about a focus (as described in the “Conceptual framework”). The terminologist also ensured that the concepts were consistently applied and accurately coded in line with the SNOMED CT guidelines [46, 47].

The participants in each focus group were given an overview of the terms and (textual) definitions discussed in their meetings to review as a final check.

#### d) Validation of the subset

The final subset, consisting of SNOMED CT patient problems with corresponding terms and definitions (*n* = 119) and associated SNOMED CT codes, was presented to all participants (*n* = 67) to determine if nursing practice was consistently covered. All the participants also confirmed that the terms and definitions accurately reflected nursing practice and that the terms used were unambiguous and understandable.

The nursing subset of SNOMED CT patient problems was also presented to the SNOMED International Nursing Special Interest Group, who were asked to review it to ensure consistency.

Review of the subset needs to be maintained over time, both to review the subset against specified use cases and to accommodate changes to existing content or add new SNOMED CT content. Separate review projects are being set up, but were beyond the scope of this study.

The final subset was distributed in an electronic format and released online. Each patient problem includes a link to a common feedback form where nurses are encouraged to make recommendations or request revisions, additions or new concepts.

### Stage 4: Distribution

Subsets can be distributed as part of the International Release, as part of a National Edition or as part of an Affiliate Edition [37]. For this study, it was decided that the subset will be distributed six-monthly as part of a National Edition, which is in line with the distribution frequency of the International Release. The standard format for distributing the SNOMED CT subset is a Simple Reference Set representing an extensional definition of a subset of components (more information about a simple reference set type can be found in the SNOMED International Practical Guide to Reference Sets [37].

### Stage 5: Implement and use

When a subset has been developed, it should be implemented for use in nursing practice. Implementation means that the subset should be integrated into software systems. It is important to support the implementation with guidance during implementation. Additionally, collaboration with users and vendors is necessary in order to test the intended use and its effectiveness. The implementation in software systems and use in practice were not included in the scope of this study and will be followed up with another study.

### Stage 6: Maintenance

This stage consisted of establishing a management and maintenance structure, including change management and the revision cycle. The management and maintenance structure was set up in line with NEN 7522:2010 nl ‘Maintenance of coding systems and other terminological systems’, which is a standard defining roles and responsibilities of organisations and people involved in the development of terminological systems. It is applicable only to Dutch healthcare [48].

## Results

### Demographics

A total of 67 participants participated in seven focus groups in order to define comprehensive, unambiguous and acceptable patient problems. The majority of participants were female (*n* = 56; 84%), which is comparable to the national proportion of nurses who are female (84%) (https://www.azwinfo.nl/; 2014). The mean age of participants was 41 (standard deviation *SD* = 12.3) – see Table 1. Compared to the national population, it is lower than average (age 43) (https://www.azwinfo.nl/; 2015). The mean length of work experience is 17 years (*SD* = 11.5). Nurses from each healthcare sector were represented in each focus group (Table 1).

**Table 1:** Demographics of the participants and focus groups

FG	N	Gender n (%)		Age mean ± SD [range]	Work experience mean ± SD [range]	Healthcare sector n (%)				
		*Male*	*Female*			*Hospital care*	*Residen-tial Care*	*Psychia-tric care*	*Primary care*	*Mentally disabled care*
1	8	3 (37%)	5 (63%)	46 ± 12.5 [23–58]	25 ± 11.8 [2–36]	3 (38%)	0	2 (25%)	2 (25%)	1 (12%)
2	15	2 (13%)	13 (87%)	42 ± 14.1 [23–60]	18 ± 12.3 [2–38]	5 (33%)	3 (20%)	3 (20%)	1 (7%)	3 (20%)
3	8	0	8 (100%)	44 ± 11.6 [27–59]	17 ± 10.7 [5–35]	2 (25%)	1 (13%)	0	4 (50%)	1 (13%)
4	8	1 (13%)	7(87%)	41 ± 10.5 [25–57]	15 ± 9.3 [2–33]	2 (25%)	1 (13%)	1 (13%)	4 (50%)	0
5	8	2 (25%)	6 (75%)	39 ± 12.7 [24–63]	13 ± 10,1 [2–30]	5 (63%)	0	1 (13%)	2 (25%)	0
6	9	1(11%)	8 (89%)	34 ± 10.9 [24–57]	11 ± 10.5 [2–34]	4 (44%)	1 (11%)	3 (33%)	0	1 (11%)
7	11	2 (18%)	9 (82%)	39 ± 12.5 [24–56]	16 ± 12.1 [2–32]	4 (36%)	5 (46%)	1 (9%)	0	1 (9%)
Total	67	11 (16%)	56 (84%)	41 ± 12.3 [23–63]	17 ± 11.5 [2–38]	25 (36%)	11 (16%)	11 (16%)	13 (19%)	7 (10%)

### Dutch nursing problem list

The resulting Dutch nursing subset of patient problems list includes 119 general patient problems labelled as a current or potential (in SNOMED CT ‘at risk’) patient problem. Each patient problem has been defined and has a SNOMED CT identifier (see Additional file 2).

Although the participants reached consensus about all concepts included, five concepts were extensively discussed prior to consensus (see Table 2). Participants felt that the proposed SNOMED CT concepts did not convey the appropriate meaning for nursing practice. These concepts were therefore excluded and replaced with the patient problem concepts in the first column of Table 2 as included in the final set. The participants have indicated that these terms reflect nursing practice properly and more understandably.

**Table 2:** Five extensively discussed concepts within the SNOMED CT core concept set

Included SNOMED CT concepts	Excluded SNOMED CT concepts
123,979,008 Abnormal body temperature (finding)	85,623,003 Ineffective thermoregulation (finding)
248,062,006 Self-injurious behaviour (finding)	130,968,006 Self-mutilation (finding)
284,905,001 Difficulty performing toileting activities (finding)	284,905,001 Self-toileting deficit (finding)
247,592,009 Poor short-term memory (finding)|	423,698,005 Limited recall of recent event (finding)
714,884,000 Difficulty transferring location (finding)	714,914,005 Impaired ability to transfer location (finding)

The participants could not find appropriate concepts to express compulsive video gaming or to express patient problems related to impaired insight into their disease, for which new concepts have been added:

□ 12,561,000,146,105 Impaired insight into the disease (finding) and.

□ 12,551,000,146,107 Compulsive video gaming (finding)

### Defining patient problems

Each patient problem was given a definition; 79 (66%) of the 119 patient problems were covered by the definitions (of the diagnosis or focus) established by the ICNP. The remaining patient problems were defined using either an official national guideline (*n* = 24; 20%) or a classification (International Classification of Functioning, Disability and Health and DSM-V) (*n* = 8; 7%). The definitions of 10 patient problems (8%) were derived from the SNOMED CT hierarchy.

### SNOMED CT identifiers

Each of the 119 patient problems has a unique SNOMED CT identifier. Of these, 65 (55%) have a matching ICNP code and 48 (40%) patient problems have a partial match. They are either more general or more detailed concepts in the SNOMED CT hierarchical structure and are not equivalent to an existing ICNP concept from the equivalency table. For example, the participants included the more general concepts ‘386,702,006 Victim of abuse (finding)’ and ‘106,143,002 Sexuality related problem (finding)’. Concepts related to abuse and sexuality are specified more precisely in ICNP. Finally, six (5%) of the 119 patient problems are not included in ICNP: obsessional thoughts, intertrigo, permanently and temporarily unable to perform work activities due to medical condition, hypomanic mood, undernourished, and disturbance in speech.

For four patient problems, the participants suspect they are included in both SNOMED CT and ICNP, but that ICNP gives a relationship with another SNOMED CT concept. For example, the SNOMED CT concept ‘Difficulty coping (finding)’ is related to ICNP’s ‘impaired adjustment’, while a concept ‘difficulty coping’ also exists in ICNP.

Three patient problems are included in both SNOMED CT and ICNP, but were not found in the SNOMED CT Equivalency Table, as shown in Table 3. According to the participants, they are equivalent.

**Table 3:** ICNP Concepts that were not incorporated in the SNOMED CT Equivalency Table for Diagnosis and Outcome Statements

SNOMED CT	ICNP
224,965,009 Grief finding (finding)	10,022,345 Grief
366,979,004 Depressed mood (finding)	10,022,402 Depressed Mood
190,902,006 Fluid imbalance (disorder)	10,042,335 Fluid imbalance

## Discussion

This study was initiated to develop a computer-comparable and exchangeable Dutch nursing subset of patient problems to assist interoperability within and between electronic health records.

The research question aimed to determine which SNOMED CT concepts covered patient problems frequently encountered in Dutch nursing practice. Together with 67 nurses, working in various Dutch healthcare settings, a total of 119 current and potential patient problems were included and defined.

Comparing the results of our study against the US nursing subset [25], there was an overlap of 55 patient problems that were included in both subsets. One possible explanation for the differences between the US subset and our Dutch subset might be that different methods were used for including patient problems. In our research, the subset is based on the overview of patient problem occurrence as experienced and the level of influence [28] in contrast to the US subset which is based on patient problems found in the Metathesaurus [25]. In addition, practicing nursing professionals were extensively involved in our study in the selection and definition of SNOMED CT concepts. Although the nursing perspective was strongly represented, in general nurses have a variety of qualification levels and are practice nurses, nurse specialists or advanced nurse practitioners. In addition, there are different views about the job descriptions and competencies of nurses. A literature study by Mistiaen et al. [49] on the role and position of professionals in the nursing profession from an international perspective not only found differences per nation in job descriptions but also in the descriptions of nursing competencies. The authors concluded that it was difficult to compare the descriptions of nursing jobs and competencies [49]. It could be that the different views on nursing competencies and tasks have influenced the selection of patient problem concepts. However, involvement of nurses in selecting and defining nursing concepts is important, because these concepts are the foundation that nurses use for planning, coordinating and evaluating nursing care and for communicating within and across healthcare settings.

The majority of the concepts (95%) either match ICNP concepts from the equivalency table or have partial matches (with an ICNP focus). This is an important finding, because the ICNP is the reference terminology for nursing. By selecting SNOMED CT concepts that match ICNP, we ensured that the SNOMED CT concepts accurately represented the nursing domain as much as possible. One interesting finding was that six concepts were not found in the ICNP. Further examination is necessary to determine if these concepts can be integrated into the ICNP.

The method used in this study not only identifies clinically relevant content for use in documentation of nursing care, but also facilitates a review process helping to harmonise both terminologies. For example, we found concepts with an equivalent ICNP concept that were not present in the equivalency table.

In this study the meanings of each patient problem concept and apparently overlapping concepts were comprehensively discussed and definitions were added. It is important to understand how patient problem concepts are structured. One of the issues that emerged was how to incorporate best evidence as outlined by clinical practice guidelines in nursing information, supported by a standardised terminology [50, 51]. Nurses are expected to apply evidence-based knowledge in their daily practice. For instance, treatment of a stage II pressure ulcer on the sacrum will be different than for a stage IV pressure ulcer stage located on the heel [52]. To ensure the best outcomes for patients, nurses need to collect and document appropriate and unambiguous information about the patient problem concept of a ‘pressure ulcer’, as outlined in a clinical practice guideline [52], such as location, stage, colour, wound edges and odour (p. 35). If this nursing information can be linked to SNOMED CT, it will not only lead to better patient outcomes and improved patient safety [53], but nurses will also be assisted in their clinical decision-making process [54, 55].

This study demonstrates that only 24 (20%) patient problems, including pressure ulcers, could be defined using the definition of an official national clinical guideline. A possible explanation for this might be either that there is no consistency between the terminology and clinical guidelines, or that few national clinical guidelines provide scientific or consensus-based evidence to deal with the patient problems that nurses come across in clinical practice. We believe it is important to take account of this issue.

### Research implications

This study has contributed to the development of computer-comparable and exchangeable information to support interoperability. This is important, because healthcare organisations are transitioning towards electronic documentation of nursing information. When organisations plan to implement the SNOMED CT nursing subset of patient problems, they may be faced with other existing nursing classifications, for instance if organisations use electronic health records based on the Omaha System. It is therefore necessary to link or associate a nursing patient problem concept to a concept from a nursing classification with the same (or similar) meaning. This process is also known as ‘mapping’. Further research should be undertaken to ensure accurate mapping.

It is also recommended that there should be international collaboration in order to establish an international nursing subset that can be used across different health systems.

### Research strengths and limitations

This study used the process model for the development of SNOMED CT subsets. Although there is only limited knowledge available about the methodological quality when developing subsets (to ensure the validity and reliability of subsets), the various stages helped structure the process and will ensure consistency for other researchers involved in developing subsets. Although this study makes an important contribution to clinical data modelling and enhances the understanding of developing terminology subsets, further research to validate the process model is recommended.

A key strength of this study is that nurses from diverse healthcare sectors were extensively involved in the development process, which is important when information is being exchanged within or between different healthcare sectors. However, nursing care takes place in a variety of healthcare settings. Nurses provide care to patients of all ages, with or without comorbidity, in different social contexts and so forth. Although we used mixed focus groups, it could be argued that not all nursing contexts were covered and consequently some patient problem concepts not have been included. This limitation may affect the extent to which the results can be generalised. However, we used the overview of patient problems (level of occurrence compared to level of reported influence) as a framework, which acted as a basis for selecting SNOMED CT concepts. This overview was set up by nurses from diverse healthcare sectors by using a pre-existing national survey panel [28]. Nevertheless, it is important to monitor the usability and completeness of the subset in different use cases.

In addition, we have gained more understanding about patient problems that are common in nursing practice and their underlying content. The findings of this study have also extended our knowledge of standardisation of nursing information and will help solve interoperability issues.

## Conclusion

The present study was designed to develop a Dutch national nursing subset of patient problems based on a standardised terminology (SNOMED CT). This study identified 119 comprehensive, unambiguous and accurately defined patient problems covering nursing practice. The study is beneficial for clinical nursing practice, because nurses will be helped by the interoperability of nursing information within and across different healthcare settings. The results also can contribute to the development of an international subset in order to investigate nursing care across nations consistently.

## Additional files

Additional file 1: Overview of patient problems (level of occurrence compared to level of reported influence). (DOCX 17 kb)

Additional file 2: Nursing subset of patient problems (DOCX 36 kb)
