# Supplementary material for: The development of a nursing subset of patient problems to support interoperability
Source: BMC Med Inform Decis Mak. 2017 Dec 4;17:158. doi: 10.1186/s12911-017-0567-5 (PMC5716238; doi:10.1186/s12911-017-0567-5)
Supplement: Supplementary file 1 — Overview of patient problems (level of occurrence compared to level of reported influence). (DOCX 17 kb) [file 12911_2017_567_MOESM1_ESM.docx]

Additional File 1: Overview of patient problems (level of occurrence compared to level of reported influence).

| **Cat** | **Frequently occurring/high level of influence experienced** | **n** | **Mean I** | **Cat** | **Frequently occurring/low level of influence experienced** | **n** | **Mean I** |
| --- | --- | --- | --- | --- | --- | --- | --- |
| 5 | Problems with defecation | 87 | 3.64 | 15 | Problems with complex interpersonal interactions, such as forming or terminating relationships | 81 | 2.96 |
| 13 | Problems washing oneself | 185 | 3.64 | 7 | Problems with functions of the joints and bones | 120 | 2.95 |
| 13 | Problems dressing | 164 | 3.64 | 4 | Problems with heart functions, including heart rate, rhythm | 130 | 294 |
| 13 | Problems toileting | 151 | 3.61 | 1 | Problems with energy and drive functions | 76 | 2.92 |
| 2 | Pain and sensation of pain | 107 | 3.54 | 1 | Problems with attention | 147 | 2.91 |
| 13 | Problems caring for parts of the body | 165 | 3.54 | 1 | Problems with temperament and personality functions | 113 | 2.9 |
| 13 | Problems eating and drinking | 97 | 3.51 | 1 | Problems with orientation functions | 137 | 2.88 |
| 5 | Problems with water, mineral and electrolyte balance functions | 81 | 3.5 | 1 | Problems with perceptual functions | 69 | 2.86 |
| 12 | Problems changing and maintaining body position | 116 | 3.45 | 4 | Problems with blood vessels | 106 | 2.8 |
| 4 | Problems with blood pressure | 131 | 3.44 | 17 | Problems with community life | 77 | 2.8 |
| 4 | Problems with the respiratory system | 104 | 3.41 | 1 | Problems with experience of self and time functions | 82 | 2.74 |
| 5 | Problems with weight maintenance | 92 | 3.39 | 1 | Problems with thought functions | 127 | 2.6 |
| 10 | Problems carrying out daily routine | 81 | 3.38 | 7 | Problems with muscle power functions | 79 | 2.57 |
| 10 | Problems undertaking a single or multiple tasks | 81 | 3.29 | 1 | Problems with memory functions | 138 | 2.53 |
| 13 | Problems with looking after own health | 164 | 3.28 | 1 | Problems with intellectual functions | 114 | 2.25 |
| 9 | Problems with problem-solving | 77 | 3.27 | **Cat** | **Less frequently occurring/low level of influence experienced** | **n** | **Mean I** |
| 12 | Moving around using transportation | 76 | 3.22 | 15 | Problems with particular interpersonal interactions, such as relating with strangers, formal relationships, family and intimate relationships | 68 | 2.95 |
| 1 | Problems with emotional functions | 167 | 3.21 | 11 | Problems with conversation | 61 | 2.93 |
| 10 | Problems handling stress and other psychological demands | 89 | 3.18 | 5 | Problems with endocrine gland functions | 30 | 2.85 |
| 12 | Problems carrying, moving and handling objects | 79 | 3.18 | 6 | Sensations associated with urinary functions | 26 | 2.84 |
| 11 | Problems communicating - receiving | 88 | 3.1 | 6 | Problems with urinary excretory functions | 42 | 2.8 |
| 12 | Problems walking and moving | 135 | 3.08 | 9 | Problems with sensory experiences | 16 | 2.8 |
| 11 | Problems communicating - producing | 74 | 3.07 | 6 | Problems with urination functions | 54 | 2.77 |
| 17 | Problems with recreation and leisure | 72 | 3.06 | 1 | Problems with consciousness | 61 | 2.75 |
| 14 | Problems with household tasks | 97 | 3.02 | 4 | Problems with functions of the immunological system | 41 | 2.62 |
| 15 | Problems with basic interpersonal interactions | 82 | 3 | 17 | Problems with religion and spirituality | 20 | 2.6 |
| 1 | Problems with sleep | 147 | 2.99 | 16 | Problems with work and employment | 38 | 2.58 |
| **Cat** | **Less frequently occurring/high level of influence experienced** | **n** | **Mean I** | 6 | Problems with sexual functions | 9 | 2.56 |
| 8 | Problems with protective functions of the skin | 44 | 3.68 | 7 | Sensations related to muscles and movement functions | 63 | 2.56 |
| 4 | Sensations associated with cardiovascular and respiratory functions | 52 | 3.5 | 16 | Problems with education | 24 | 2.55 |
| 5 | Problems with thermoregulatory functions | 43 | 3.46 | 14 | Problems finding a place to live | 29 | 2.52 |
| 6 | Sensations associated with genital and reproductive functions | 5 | 3.4 | 16 | Problems with economic life | 43 | 2.49 |
| 8 | Problems with functions of the hair and nails | 14 | 3.38 | 2 | Problems with hearing | 60 | 2.44 |
| 8 | Problems with repair functions of the skin | 28 | 3.33 | 7 | Problems with muscle endurance | 21 | 2.42 |
| 5 | Problems with ingestion | 49 | 3.29 | 6 | Problems with menstruation | 5 | 2.4 |
| 5 | Problems with functions related to the metabolic system | 58 | 3.23 | 9 | Problems with basic learning and applying knowledge | 37 | 2.39 |
| 11 | Problems with communication devices and techniques | 13 | 3.18 | 7 | Problems with muscle tone | 51 | 2.36 |
| 5 | Sensations associated with the digestive system, including nausea, feeling bloated etc. | 56 | 3.16 | 2 | Problems with taste, smell and touch | 43 | 2.3 |
| 8 | Sensation related to the skin | 23 | 3.14 | 6 | Problems with procreation functions | 4 | 2.25 |
| 5 | Problems with digestive functions | 28 | 3.04 | 7 | Problems with involuntary movement | 31 | 2.2 |
| 14 | Problems shopping and gathering daily necessities | 65 | 3.03 | 2 | Problems seeing | 45 | 2.17 |
| 4 | Problems with functions of the haematological system | 58 | 3 | 3 | Problems with the voice | 20 | 1.95 |
|  |  |  |  | 3 | Problems with fluency and rhythm of speech | 18 | 1.94 |
|  |  |  |  | 3 | Problems with articulation | 31 | 1.83 |
