# Supplementary material for: The development of a nursing subset of patient problems to support interoperability
Source: BMC Med Inform Decis Mak. 2017 Dec 4;17:158. doi: 10.1186/s12911-017-0567-5 (PMC5716238; doi:10.1186/s12911-017-0567-5)
Supplement: Supplementary file 2 — Nursing subset of patient problems (DOCX 36 kb) [file 12911_2017_567_MOESM2_ESM.docx]

Additional File 2: Nursing subset of patient problems

| **Snomed FSN** | **Text definition** | **Snomed ID** | **Match ICNP concept** | **Partial match with ICNP concept** | **No match with ICNP concept** |
| --- | --- | --- | --- | --- | --- |
| abnormal body temperature (finding) | problem with the body temperature, measured in ° C (degrees Celsius). Body temperature is defined by 'internal body heat related to body metabolism' (ICNP) | [123979008](https://terminologie.nictiz.nl/terminology/snomed/viewConcept/123979008) |  | 1 |  |
| abnormal defecation (finding) | problem with movement and evacuation of stool through the bowel (ICNP) | [179950008](https://terminologie.nictiz.nl/terminology/snomed/viewConcept/179950008) |  | 1 |  |
| acute confusion (finding) | image of acute and chronic change, often fluctuating which includes attention deficit disorder and consciousness and changes in cognition (memory, orientation, language) or observation (guideline delirium adults and elderly; NVKG 2013) | [130987000](https://terminologie.nictiz.nl/terminology/snomed/viewConcept/130987000) | 1 |  |  |
| acute pain (finding) | pain is an unpleasant sensory and emotional experience associated with actual or potential tissue damage or described in terms of such damage (Guideline postoperative pain; 2012) | [274663001](https://terminologie.nictiz.nl/terminology/snomed/viewConcept/274663001) | 1 |  |  |
| alcohol abuse (disorder) | misuse of alcohol for a non-therapeutic effect that may be harmful to health and may cause addiction (ICNP) | [15167005](https://terminologie.nictiz.nl/terminology/snomed/viewConcept/15167005) | 1 |  |  |
| altered body image (finding) | problem with the mental picture of one's own body in whole or in part, or of one's physical appearance (ICNP) | [225507001](https://terminologie.nictiz.nl/terminology/snomed/viewConcept/225507001) | 1 |  |  |
| anxiety (finding) | a sense of threat, danger or stress, without concrete content and not related to an object (ICNP) | [48694002](https://terminologie.nictiz.nl/terminology/snomed/viewConcept/48694002) | 1 |  |  |
| aphasia (finding) | defective or absent language function of using and understanding words together with damage to certain parts of the brain (ICNP) | [87486003](https://terminologie.nictiz.nl/terminology/snomed/viewConcept/87486003) |  | 1 |  |
| at risk for falls (finding) | an unintended change of the body position, which results in landing on the floor or any other lower level (Guideline prevention of fall incidients of elderly; CBO,2004) | [129839007](https://terminologie.nictiz.nl/terminology/snomed/viewConcept/129839007) | 1 |  |  |
| at risk for infection (finding) | risk for infection. Infection is defined by 'an invasion of the body by pathogenic microorganisms that reproduce and multiply, causing disease by local cellular injury, secretion of toxin or antigen-antibody reaction' (ICNP) | [78648007](https://terminologie.nictiz.nl/terminology/snomed/viewConcept/78648007) | 1 |  |  |
| at risk for suicide (finding) | risk for suicide. Suicide is defined by 'performing suicidal activities which lead to death of self' (ICNP) | [225444004](https://terminologie.nictiz.nl/terminology/snomed/viewConcept/225444004) | 1 |  |  |
| at risk of acute confusion (finding) | image of acute and chronic change, often fluctuating which includes attention deficit disorder and consciousness and changes in cognition (memory, orientation, language) or observation (guideline delirium adults and elderly; NVKG 2013) | [423192007](https://terminologie.nictiz.nl/terminology/snomed/viewConcept/423192007) | 1 |  |  |
| at risk of constipation (finding) | risk for decrease in the frequency of defecation accompanied by difficulty or incomplete passage of stool; passage of excessively hard, dry stool (ICNP) | [129691005](https://terminologie.nictiz.nl/terminology/snomed/viewConcept/129691005) | 1 |  |  |
| at risk of loneliness (finding) | at risk for the subjective experience of a pleasurable or unacceptable lack of (quality of) certain social relationships (De Jong Gierveld, Tilburg; 2007/Platform ouderenzorg) | [129698004](https://terminologie.nictiz.nl/terminology/snomed/viewConcept/129698004) | 1 |  |  |
| at risk of pressure ulcer (finding) | at risk for a localized damage to the skin and/or underlying tissue, mostly at the level of a bony prominence, as a result of pressure or pressure in combination with sliding force (National multidisciplinary guideline pressure ulcer prevention and treatment; V&VN, nov 2011) | [285304000](https://terminologie.nictiz.nl/terminology/snomed/viewConcept/285304000) | 1 |  |  |
| at risk of undernutrition (finding) | at risk for a nutritional status in which there is a deficiency or imbalance of energy, protein, and/or other nutrients, which leads to measurable adverse effects on the body size and body composition, on the functioning and on clinical outcomes (Guideline screening and treatment of undernutrition, stuurgroep ondervoeding; juni 2011) | [129845004](https://terminologie.nictiz.nl/terminology/snomed/viewConcept/129845004) | 1 |  |  |
| blood pressure alteration (finding) | problem with effects of fluctuating blood pressure. Bloodpressure is defined by 'the force of circulating blood on blood vessel walls, or the hydrostatic pressure exerted by the blood on the vasculature of the arteries' (ICNP) | [129899009](https://terminologie.nictiz.nl/terminology/snomed/viewConcept/129899009) | 1 |  |  |
| body temperature above reference range (finding) | decreased ability to change internal thermostat accompanied by increased body temperature, warm dry skin, drowsiness and headache associated with dysfunction of the central nervous system or endocrine system (ICNP) | [50177009](https://terminologie.nictiz.nl/terminology/snomed/viewConcept/50177009) | 1 |  |  |
| burn of skin (disorder) | an injury due to the influence of heat on the skin for a certain period of time and above a certain critical temperature, above the critical temperature (+/- 40° C) occurs damage to the skin (Association of Dutch Burn Centres http://brandwondenstichting.nl/brandwonden-voorkomen/wat-zijn-brandwonden/) | [284196006](https://terminologie.nictiz.nl/terminology/snomed/viewConcept/284196006) |  | 1 |  |
| caregiver difficulty performing caretaking (finding) | problem with personal care; unpaid and often long-term care for sick family members or friends (more than eight hours per week and/or longer than three months) (https://www.rijksoverheid.nl/onderwerpen/mantelzorg) | [705044006](https://terminologie.nictiz.nl/terminology/snomed/viewConcept/705044006) | 1 |  |  |
| chronic pain (finding) | pain is an unpleasant sensory and emotional experience associated with actual or potential tissue damage or described in terms of such damage (Concept care standard chronic pain; 2016) | 82423001 | 1 |  |  |
| obsessional thoughts (finding) | persistent thought or idea that the mind is constantly and involuntarily working on (http://www.psychischegezondheid.nl/) | [67698009](https://terminologie.nictiz.nl/terminology/snomed/viewConcept/67698009) |  | 1 |  |
| compulsive gambling (disorder) | extensively gambling with an effect that may be harmful to health (ICNP) | [18085000](https://terminologie.nictiz.nl/terminology/snomed/viewConcept/18085000) |  | 1 |  |
| compulsive video gaming (disorder) | extensively gaming with an effect that may be harmful to health (ICNP) | [12551000146107](https://terminologie.nictiz.nl/terminology/snomed/viewConcept/12551000146107) |  | 1 |  |
| delusions (finding) | false sense of reality that cannot be corrected by reason, argument or persuasion or by evidence of one's own senses (ICNP) | [2073000](https://terminologie.nictiz.nl/terminology/snomed/viewConcept/2073000) |  | 1 |  |
| depressed mood (finding) | feelings from sadness to melancholy with decreased concentration, loss of appetite and insomnia (ICNP) | [366979004](https://terminologie.nictiz.nl/terminology/snomed/viewConcept/366979004) | 1 |  |  |
| diarrhea (finding) | Passage of loose, liquid, unformed stool, increased frequency of elimination accompanied by increased bowel sounds, cramping and urgency of defecation (ICNP) | [62315008](https://terminologie.nictiz.nl/terminology/snomed/viewConcept/62315008) | 1 |  |  |
| difficulty changing position (finding) | problem with getting into and out of a body position and moving from one location to another, such as getting up out of a chair to lie down on a bed, and getting into and out of positions of kneeling or squatting [ICF] | [303387009](https://terminologie.nictiz.nl/terminology/snomed/viewConcept/303387009) |  | 1 |  |
| difficulty complying with treatment (finding) | problem with performing activities to meet therapeutic health care requirements and conform to the prescribed treatment course of the caregiver and the healthcare provider (Time for better use of medicines; NPCF, 2008) | [304900007](https://terminologie.nictiz.nl/terminology/snomed/viewConcept/304900007) |  | 1 |  |
| difficulty coping (finding) | problem with managing stress and having a sense of control and increased psychological comfort (ICNP) | [18232000](https://terminologie.nictiz.nl/terminology/snomed/viewConcept/18232000) | 1 |  |  |
| difficulty drinking (finding) | problem with taking fluids during meals and during the day or when thirsty (ICNP) | [288857007](https://terminologie.nictiz.nl/terminology/snomed/viewConcept/288857007) |  | 1 |  |
| difficulty establishing relationships (finding) | difficulty in starting an engagement with one or more persons (ICNP) | [423238001](https://terminologie.nictiz.nl/terminology/snomed/viewConcept/423238001) |  | 1 |  |
| self-feeding deficit (finding) | problem with bringing food to the mouth and feeding oneself until satisfied (ICNP) | [7653001](https://terminologie.nictiz.nl/terminology/snomed/viewConcept/7653001) | 1 |  |  |
| difficulty inferring meaning (finding) | problem to understand information with the mind, both verbally and nonverbally [ICF] | [310825003](https://terminologie.nictiz.nl/terminology/snomed/viewConcept/310825003) |  | 1 |  |
| difficulty maintaining a position (finding) | staying in the same body position as required, such as remaining seated or remaining standing  [ICF] | [282851000](https://terminologie.nictiz.nl/terminology/snomed/viewConcept/282851000) |  | 1 |  |
| difficulty maintaining relationships (finding) | problem maintaining contact within a relationship with one or more persons under the social rules in the company (ICNP) | [424573006](https://terminologie.nictiz.nl/terminology/snomed/viewConcept/424573006) |  | 1 |  |
| difficulty making conversation (finding) | problem with making a conversation or verbal communication between two or more persons (SNOMDED CT hierarchy) | [288638001](https://terminologie.nictiz.nl/terminology/snomed/viewConcept/288638001) |  | 1 |  |
| difficulty managing medication (finding) | problem with the independently use and administration of medication (ICNP) | [285038001](https://terminologie.nictiz.nl/terminology/snomed/viewConcept/285038001) | 1 |  |  |
| difficulty managing personal financial activities (finding) | problem with caring for and managing personal money/capital (ICNP) | [300686005](https://terminologie.nictiz.nl/terminology/snomed/viewConcept/300686005) | 1 |  |  |
| difficulty performing dressing activities (finding) | problem with putting on or removing clothes (ICNP) | [284972002](https://terminologie.nictiz.nl/terminology/snomed/viewConcept/284972002) | 1 |  |  |
| difficulty performing mouthcare activities (finding) | problem taking care of mouth and teeth/molars or dentures (SNOMED CT hierarchy) | [289128002](https://terminologie.nictiz.nl/terminology/snomed/viewConcept/289128002) | 1 |  |  |
| difficulty performing personal grooming activity (finding) | problem with brushing and in other ways look after and clean the hair and nails (ICNP) | [704439001](https://terminologie.nictiz.nl/terminology/snomed/viewConcept/704439001) | 1 |  |  |
| difficulty performing shopping activities (finding) | problem with buying items necessary for maintaining daily life; purchasing, trading or bartering for items needed for the home (ICNP) | [300723007](https://terminologie.nictiz.nl/terminology/snomed/viewConcept/300723007) | 1 |  |  |
| difficulty performing toileting activities (finding) | problem with carrying out toileting activities for urination and defecation (ICNP) and take care of oneselves | [284905001](https://terminologie.nictiz.nl/terminology/snomed/viewConcept/284905001) |  | 1 |  |
| difficulty performing washing and drying activities (finding) | problem with the washing and drying of the entire body, or body parts, such as bathing, showering, washing of hands and feet, face and hair (ICNP) | [288552005](https://terminologie.nictiz.nl/terminology/snomed/viewConcept/288552005) |  | 1 |  |
| difficulty preparing food for eating (finding) | problem with taking care of providing food and meals in terms of quantity and quality, processing of food, storing of food, serving and distribution of food necessary for maintaining daily life (ICNP) | [286457005](https://terminologie.nictiz.nl/terminology/snomed/viewConcept/286457005) | 1 |  |  |
| difficulty transferring location (finding) | problem with moving from one surface to another, such as sliding along a bench or moving from a bed to a chair, without changing body position [ICF] | [714884000](https://terminologie.nictiz.nl/terminology/snomed/viewConcept/714884000) |  | 1 |  |
| difficulty using self-expression (finding) | problem with specific mental functions necessary to produce meaningful messages in spoken, written, signed or other forms of language [ICF] | [288742009](https://terminologie.nictiz.nl/terminology/snomed/viewConcept/288742009) |  | 1 |  |
| disorientated (finding) | problem with ascertaining relationship to environment in terms of time; in terms of place at a given point in time and in terms of awareness of own identity and in terms of recognition of people around (ICNP) | [62476001](https://terminologie.nictiz.nl/terminology/snomed/viewConcept/62476001) | 1 |  |  |
| disorientated in place (finding) | problem with ascertaining relationship to environment in terms of place at a given point in time such as country, province, city, workplace, home (ICNP) | [72440003](https://terminologie.nictiz.nl/terminology/snomed/viewConcept/72440003) |  | 1 |  |
| disorientated in time (finding) | problem with ascertaining relationship to environment in terms of time such as year, season, month, day, precise time (ICNP) | [19657006](https://terminologie.nictiz.nl/terminology/snomed/viewConcept/19657006) |  | 1 |  |
| disorientation for person (finding) | problem with ascertaining relationship to environment in terms of awareness of own identity such as age, date of birth and in terms of recognition of people around (ICNP) | [62766000](https://terminologie.nictiz.nl/terminology/snomed/viewConcept/62766000) |  | 1 |  |
| disturbance in role performance (finding) | problem with interacting according to implicit or explicit set of expectations, rules and standards of behaviour expected by others (ICNP) | [27179007](https://terminologie.nictiz.nl/terminology/snomed/viewConcept/27179007) | 1 |  |  |
| disturbance in speech (finding) | problem with the production of various sounds by the passage of air through the larynx [ICF] | [29164008](https://terminologie.nictiz.nl/terminology/snomed/viewConcept/29164008) |  |  | 1 |
| disturbance of attention (finding) | focused attention and mental activity to store and recall knowledge (ICNP) | [76039005](https://terminologie.nictiz.nl/terminology/snomed/viewConcept/76039005) |  | 1 |  |
| disturbance of consciousness (finding) | problem with mental responsiveness to impressions from a combination of the senses, keeping the mind alert and sensitive to the external environment (ICNP) | [3006004](https://terminologie.nictiz.nl/terminology/snomed/viewConcept/3006004) |  | 1 |  |
| diversional activity deficit (finding) | problem with spending time on diversional activities like hobbies, sport, recreation (SNOMED CT hierarchy) | [23935006](https://terminologie.nictiz.nl/terminology/snomed/viewConcept/23935006) | 1 |  |  |
| drug abuse (disorder) | misuse of drugs or medication for a non-therapeutic effect that may be harmful to health and may cause addiction (ICNP) | [26416006](https://terminologie.nictiz.nl/terminology/snomed/viewConcept/26416006) | 1 |  |  |
| dyspnea (finding) | conscious experience of a disruption of breathing, in other words the feeling of inadequate breathing, an unpleasant and particularly threatening and anxious feeling. The degree of the experienced dyspnea is not dependent on the severity of the underlying cause (Guideline dyspnoe, cough and rattle; IKZ, 2005; www.oncoline.nl) | [267036007](https://terminologie.nictiz.nl/terminology/snomed/viewConcept/267036007) | 1 |  |  |
| eczema (disorder) | itchy, polymorphic skin with redness, edema, papules, vesicles, crusts, flakes and/or lichenification, as a result of a non-infectious inflammation of the skin, caused by intrinsic and/or environmental factors (https://www.nhg.org/standaarden/samenvatting/eczeem#idp19440) | [43116000](https://terminologie.nictiz.nl/terminology/snomed/viewConcept/43116000) |  | 1 |  |
| eruption of skin (disorder) | skin eruption of erythema of different colours and protuberance, local oedema, urticaria, vesicles and itching (ICNP) | [271807003](https://terminologie.nictiz.nl/terminology/snomed/viewConcept/271807003) |  | 1 |  |
| fatigue (finding) | feelings of decreased strength or endurance, weariness, mental or physical tiredness, and listlessness with lower capacity of physical or mental work (ICNP) | [84229001](https://terminologie.nictiz.nl/terminology/snomed/viewConcept/84229001) | 1 |  |  |
| feeling agitated (finding) | condition of purposeless psychomotor excitement, restless activity, pacing, releasing of nervous tension associated with anxiety, fear or mental stress (ICNP) | [24199005](https://terminologie.nictiz.nl/terminology/snomed/viewConcept/24199005) | 1 |  |  |
| feeling lonely (finding) | the subjective experience of a pleasurable or unacceptable lack of (quality of) certain social relationships (De Jong Gierveld, Tilburg; 2007/Platform ouderenzorg) | [267076002](https://terminologie.nictiz.nl/terminology/snomed/viewConcept/267076002) |  | 1 |  |
| fertility problem (finding) | problem with capacity to participate in the conception of a live foetus that delivers as a viable child (ICNP) | [27034006](https://terminologie.nictiz.nl/terminology/snomed/viewConcept/27034006) |  | 1 |  |
| finding of speed of thought (finding) | problem with an accelerated or slowed thought process (SNOMED CT hierarchy) | [365276007](https://terminologie.nictiz.nl/terminology/snomed/viewConcept/365276007) |  | 1 |  |
| finding related to risk factor in pregnancy (finding) | risk of complications during pregnancy. Pregnany is the condition of growing and nurturing a developing foetus in body lasting from approximately 266 days period to birth from the day of fertilisation (ICNP) | [199312002](https://terminologie.nictiz.nl/terminology/snomed/viewConcept/199312002) | 1 |  |  |
| fine motor impairment (finding) | problem with the use of the hands and fingers for small movements, the grasping and manipulation of an object (http://www.fijne-motoriek.nl/) | [228148009](https://terminologie.nictiz.nl/terminology/snomed/viewConcept/228148009) |  | 1 |  |
| fluid imbalance (disorder) | regulation of uptake and excretion of body fluids such as the quantity and balance of water and electrolytes in the body (ICNP) | [190902006](https://terminologie.nictiz.nl/terminology/snomed/viewConcept/190902006) | 1 |  |  |
| grief finding (finding) | feelings of sorrow associated with anticipatory or actual significant loss and death; Shock and disbelief, (Stage of Shock); Exhaustion, extreme tiredness and lethargy, mental anguish, reactions of bereavement and mourning, crying or sobbing, alarm, disbelief, denial, anger (Stage of Reaction); Adjustment, acceptance, reorientation, express feelings of loss, accept reality of loss, absence of somatic stress, express positive expectations about the future (Stage of acceptance) (ICNP) | [224965009](https://terminologie.nictiz.nl/terminology/snomed/viewConcept/224965009) |  | 1 |  |
| hallucinations (finding) | apparent registration of sensory stimuli which are not actually present, classified according to the senses, such as auditory, visual, olfactory, gustatory or tactile hallucinations (ICNP) | [7011001](https://terminologie.nictiz.nl/terminology/snomed/viewConcept/7011001) | 1 |  |  |
| hearing problem (finding) | problem with faculty of hearing due to responses to stimuli from auditory organs, capacity to hear (ICNP) | [300228004](https://terminologie.nictiz.nl/terminology/snomed/viewConcept/300228004) | 1 |  |  |
| housing problems (finding) | problem with the residential building, constructed for human dwelling, residence and homes, shelter providing protections and space for humans, such as unsuited to limitations in mobility, poor housing, lack of space, moisture in the house (ICNP) | [105531004](https://terminologie.nictiz.nl/terminology/snomed/viewConcept/105531004) |  | 1 |  |
| hypomanic mood (finding) | a distinct period of abnormally and persistently elevated, expansive, or irritable mood [DSM-V] | [281257007](https://terminologie.nictiz.nl/terminology/snomed/viewConcept/281257007) |  |  | 1 |
| hypothermia (finding) | decreased ability to change internal thermostat, reduced body temperature, cool, pale and dry skin, shivering, slow capillary refill, tachycardia, cyanotic nail beds, hypertension, piloerection associated with prolonged exposure to cold, dysfunction of the central nervous system or endocrine system (ICNP) | [386689009](https://terminologie.nictiz.nl/terminology/snomed/viewConcept/386689009) | 1 |  |  |
| impaired ability to learn new material (finding) | problem with the process of acquiring knowledge or skill by means of systematic study, instruction, practice, training or experience (ICNP) | [247617009](https://terminologie.nictiz.nl/terminology/snomed/viewConcept/247617009) |  | 1 |  |
| impaired home maintenance management (finding) | problem with practice of care for or proper attention to making environment or dwelling place comfortable, cosy; making oneself and others feel at home; providing a secure and well managed household (ICNP) | [67175002](https://terminologie.nictiz.nl/terminology/snomed/viewConcept/67175002) | 1 |  |  |
| impaired insight in disease | problem with the realization that (or understanding that) a person has a disease or health problem, and what the significance for life is (http://mens-en-gezondheid.infonu.nl/ziekten/82477-ziekte-ziektebesef-en-betekenis.html) | [12561000146105](https://terminologie.nictiz.nl/terminology/snomed/viewConcept/12561000146105) |  | 1 |  |
| impaired social interaction (finding) | problem with behavior of mutual social interchange, participation and social exchange among individuals and groups (ICNP) | [88598008](https://terminologie.nictiz.nl/terminology/snomed/viewConcept/88598008) | 1 |  |  |
| impaired touch discrimination (finding) | problem with the faculty of feeling due+ to responses to stimuli from tactile organs, capacity for orientation by touch and pressure from tactile organs in integument (ICNP) | [299923005](https://terminologie.nictiz.nl/terminology/snomed/viewConcept/299923005) |  | 1 |  |
| impairment of mental alertness (finding) | problem with the level of watchfulness or vigilance, paying attention to something, ready to take action (ICNP) | [704426000](https://terminologie.nictiz.nl/terminology/snomed/viewConcept/704426000) | 1 |  |  |
| inadequate social support (finding) | lack of social or psychological help from somebody to succeed, keep from failing and to bear the weight of and maintain in position, hold up [ICNP] | [425022003](https://terminologie.nictiz.nl/terminology/snomed/viewConcept/425022003) | 1 |  |  |
| incontinence of feces (finding) | involuntary, uncontrolled passage and expulsion of stool (ICNP) | [72042002](https://terminologie.nictiz.nl/terminology/snomed/viewConcept/72042002) | 1 |  |  |
| ineffective breathing pattern (finding) | difficulty with pattern of moving air into and out of the lungs with a certain respiratory rate and rhythm, depth of inspiration and strength of expiration [ICNP, indirect] | [20573003](https://terminologie.nictiz.nl/terminology/snomed/viewConcept/20573003) |  | 1 |  |
| intertrigo (disorder) | a localized superficial skin disorder in the large skin folds,  which is characterized by continued redness (erythema) on both sides of the fold. In addition,  one or more of the following symptoms can occur: maceration (softening), fissures (cracks), erosions, a oozing skin or crust formation (National multidisciplinary guideline (intertrigo) prevention and treatment; V&VN, 2011) | [58759008](https://terminologie.nictiz.nl/terminology/snomed/viewConcept/58759008) |  |  | 1 |
| itching of skin (finding) | sensation of annoying tingling, cutaneous feeling followed by impulse to scratch skin (ICNP) | [418363000](https://terminologie.nictiz.nl/terminology/snomed/viewConcept/418363000) | 1 |  |  |
| low self-control (finding) | problem with disposition taken to take care of what is needed to maintain oneself, keeping oneself going, handle basic individual and intimate necessities and activities in life (ICNP) | [705000008](https://terminologie.nictiz.nl/terminology/snomed/viewConcept/705000008) | 1 |  |  |
| manic mood (finding) | continued abnormally elevated disinhibited state of emotion/loss of inhibition, filled with positive vitality, negating concerns and decreased need for sleep [DSM-V] | [405273008](https://terminologie.nictiz.nl/terminology/snomed/viewConcept/405273008) |  | 1 |  |
| memory impairment (finding) | problem with mental acts by which sensations, impressions and ideas are stored and recalled (ICNP) | [386807006](https://terminologie.nictiz.nl/terminology/snomed/viewConcept/386807006) | 1 |  |  |
| menstruation finding (finding) | problem with the recurring cycle of shedding, re-growth and proliferating of the endometrium of the uterus as menstruation; average length of the menstrual cycle from first day of bleeding to first of another is 28 days; length; duration and quantity vary; menstrual cycle begins at menarche and ends at the menopause (ICNP) | [32301000146106](https://terminologie.nictiz.nl/terminology/snomed/viewConcept/32301000146106) |  | 1 |  |
| mood swings (finding) | problem with a varying level of feelings and emotional tone (ICNP) | [18963009](https://terminologie.nictiz.nl/terminology/snomed/viewConcept/18963009) | 1 |  |  |
| nausea (finding) | sensation of feeling sick with an inclination to vomit, unpleasant sensation vaguely referred to the epigastrium and abdomen, offensive to taste or smell (ICNP) | [422587007](https://terminologie.nictiz.nl/terminology/snomed/viewConcept/422587007) | 1 |  |  |
| obstipation (disorder) | decrease in the frequency of defecation accompanied by difficulty or incomplete passage of stool; passage of excessively hard, dry stool (ICNP) | [14760008](https://terminologie.nictiz.nl/terminology/snomed/viewConcept/14760008) | 1 |  |  |
| overweight (finding) | overweight among adults (18-70 years) exists as a body mass index (BMI) ≥25 (with BMI ≥30 obesity) in children is overweight diagnosed on the basis of four criteria: physique, ethnicity, puberty and fat distribution (http://www.voedingscentrum.nl/nl.aspx) | [238131007](https://terminologie.nictiz.nl/terminology/snomed/viewConcept/238131007) | 1 |  |  |
| pain (finding) | an unpleasant sensory and emotional experience, where the experience of pain is what a person experiencing the pain says it is and and is present whenever he/she says that it's present (NHG-Werkgroep Pijn. NHG-Standard pain. Primary care law 2015;58(9):472-85) | [22253000](https://terminologie.nictiz.nl/terminology/snomed/viewConcept/22253000) | 1 |  |  |
| permanently unable to perform work activities due to medical condition (finding) | ongoing problem with the extent and manner in which people participate in work (SNOMED CT hierarchy) | [440584001](https://terminologie.nictiz.nl/terminology/snomed/viewConcept/440584001) |  |  | 1 |
| physical aggression (finding) | forceful demonstration of actions or unjust use of force or power with the purpose to injure or damage, mistreat or assault: Violent, assaulting, harmful, illegal or cultural prohibited actions toward something or someone else; state of power struggle or conflict (ICNP) | [248004009](https://terminologie.nictiz.nl/terminology/snomed/viewConcept/248004009) | 1 |  |  |
| poor long-term memory (finding) | problem with the ability to recall or remember past events or experiences (ICNP) | [247588002](https://terminologie.nictiz.nl/terminology/snomed/viewConcept/247588002) |  | 1 |  |
| poor short-term memory (finding) | problem with the ability to recall or remember recent events or experiences (ICNP) | [247592009](https://terminologie.nictiz.nl/terminology/snomed/viewConcept/247592009) |  | 1 |  |
| preoccupation (finding) | dominating and engrossing the mind to the exclusion of other thoughts or being mentally distracted (ICNP) | [247632002](https://terminologie.nictiz.nl/terminology/snomed/viewConcept/247632002) |  | 1 |  |
| pressure ulcer (disorder) | a localized damage to the skin and/or underlying tissue, mostly at the level of a bony prominence, as a result of pressure or pressure in combination with sliding force (National multidisciplinary guideline pressure ulcer prevention and treatment; V&VN, nov 2011) | [399912005](https://terminologie.nictiz.nl/terminology/snomed/viewConcept/399912005) | 1 |  |  |
| retention of urine (disorder) | involuntary accumulation of urine in bladder, incomplete emptying of bladder associated with a loss of muscle function in bladder, side effects of narcotics or damage to bladder (ICNP) | [267064002](https://terminologie.nictiz.nl/terminology/snomed/viewConcept/267064002) | 1 |  |  |
| self-injurious behavior (finding) | performing of self initiated activities with the purpose of hurting or damaging oneself, violence directed towards oneself (ICNP) | [248062006](https://terminologie.nictiz.nl/terminology/snomed/viewConcept/248062006) | 1 |  |  |
| sense of smell impaired (finding) | problem with the faculty of smelling due to responses to stimuli from olfactory organs, capacity to smell odours (ICNP) | [83156004](https://terminologie.nictiz.nl/terminology/snomed/viewConcept/83156004) | 1 |  |  |
| sexuality related problem (finding) | problem with the ability of participating in intimity and sexual intercourse; Sexuality includes all feelings, thoughts, beliefs, fantasies, desires and behaviors that are sexually oriented (ICNP) | [106143002](https://terminologie.nictiz.nl/terminology/snomed/viewConcept/106143002) |  | 1 |  |
| sleep pattern disturbance (finding) | problem with sleeping. Sleeping is defined by 'Recurring lowering of bodily activity marked by reduced consciousness, not awake accompanied with, not aware, depressed metabolism, immobile posture, diminished bodily activity, diminished but readily reversible sensitivity to external stimuli' (ICNP) | [26677001](https://terminologie.nictiz.nl/terminology/snomed/viewConcept/26677001) | 1 |  |  |
| swallowing problem (finding) | problem with the passage of fluids and decomposed food from mouth by movement of tongue and muscles through throat and oesophagus to stomach (ICNP) | [399122003](https://terminologie.nictiz.nl/terminology/snomed/viewConcept/399122003) | 1 |  |  |
| taste sense altered (finding) | problem with the faculty of t+asting due to responses to stimuli from gustatory organs, capacity to taste food and drink (ICNP) | [271801002](https://terminologie.nictiz.nl/terminology/snomed/viewConcept/271801002) | 1 |  |  |
| temporarily unable to perform work activities due to medical condition (finding) | temporarily problem with the extent and manner in which people participate in work (SNOMED CT hierarchy) | [440337002](https://terminologie.nictiz.nl/terminology/snomed/viewConcept/440337002) |  |  | 1 |
| tobacco dependence syndrome (disorder) | misuse of tobacco for a non-therapeutic effect that may be harmful to health and may cause addiction (ICNP) | [89765005](https://terminologie.nictiz.nl/terminology/snomed/viewConcept/89765005) |  | 1 |  |
| undernourished (finding) | nutritional status in which there is a deficiency or imbalance of energy, protein, and/or other nutrients, which leads to measurable adverse effects on the body size and body composition, on the functioning and on clinical outcomes (Guideline screening and treatment of undernutrition, stuurgroep ondervoeding; juni 2011) | [248325000](https://terminologie.nictiz.nl/terminology/snomed/viewConcept/248325000) |  |  | 1 |
| underweight (finding) | ‘Body Mass index’ (BMI) ≤18,5 (patients ≥65 year: BMI ≤20) (Guideline screening and treatment of undernutrition, stuurgroep ondervoeding; juni 2011) | [248342006](https://terminologie.nictiz.nl/terminology/snomed/viewConcept/248342006) | 1 |  |  |
| urinary incontinence (finding) | involuntary passage of urine, failure of voluntary control over bladder and urethral sphincter (ICNP) | [165232002](https://terminologie.nictiz.nl/terminology/snomed/viewConcept/165232002) | 1 |  |  |
| verbal aggression (finding) | forceful, self-assertive action or attitude expressed verbally, physically or symbolically (ICNP) | [248003003](https://terminologie.nictiz.nl/terminology/snomed/viewConcept/248003003) |  | 1 |  |
| victim of abuse (finding) | victim of acts of physical, emotional and sexual assault, such as rape and mistreatment (ICNP beta 2) | [386702006](https://terminologie.nictiz.nl/terminology/snomed/viewConcept/386702006) |  | 1 |  |
| visual impairment (disorder) | problem with the ability to see as a result of response to stimuli of visual organs (SNOMED CT hierarchy) | [7973008](https://terminologie.nictiz.nl/terminology/snomed/viewConcept/7973008) |  | 1 |  |
| vomiting (disorder) | expulsion or bringing up of converted food or stomach content through the oesophagus and out of mouth (ICNP) | [422400008](https://terminologie.nictiz.nl/terminology/snomed/viewConcept/422400008) | 1 |  |  |
| walking disability (finding) | problem with moving body from one place to another by moving legs stepwise by self, capacity to bear weight of body and walk with effective gait within the range of speed from slow, moderate and fast pace, upstairs, downstairs, up inclines and down inclines (ICNP) | [228158008](https://terminologie.nictiz.nl/terminology/snomed/viewConcept/228158008) | 1 |  |  |
| wound of skin (disorder) | an interruption in the continuity of the skin, usually caused by external influences. Injury of the tissue, usually associated with physical or mechanical damage; sloughing and tunneling of the tissue ( Guideline wound care, vereniging voor heelkunde; 2013) | [262526004](https://terminologie.nictiz.nl/terminology/snomed/viewConcept/262526004) |  | 1 |  |
| **Total patient problems: 119** |  |  | **65** | **48** | **6** |
